# Decreased Polysaccharide Feruloylation Compromises Plant Cell Wall Integrity and Increases Susceptibility to Necrotrophic Fungal Pathogens

**DOI:** 10.3389/fpls.2016.00630

**Published:** 2016-05-10

**Authors:** Nathan T. Reem, Gennady Pogorelko, Vincenzo Lionetti, Lauran Chambers, Michael A. Held, Daniela Bellincampi, Olga A. Zabotina

**Affiliations:** ^1^Roy J. Carver Department of Biochemistry, Biophysiscs and Molecular Biology, Iowa State University, Ames, IAUSA; ^2^Dipartmento di Biologia e Biotechnologie “Charles Darwin,” Sapienza Universita di Roma, RomeItaly; ^3^Department of Chemistry and Biochemistry, Ohio University, Athens, OHUSA

**Keywords:** ferulic acid, cell wall integrity, *Arabidopsis thaliana*, *Brachypodium distachyon*, plant pathogen resistance

## Abstract

The complexity of cell wall composition and structure determines the strength, flexibility, and function of the primary cell wall in plants. However, the contribution of the various components to cell wall integrity (CWI) and function remains unclear. Modifications of cell wall composition can induce plant responses known as CWI control. In this study, we used transgenic expression of the fungal feruloyl esterase *AnFAE* to examine the effect of post-synthetic modification of *Arabidopsis* and *Brachypodium* cell walls. Transgenic *Arabidopsis* plants expressing *AnFAE* showed a significant reduction of monomeric ferulic acid, decreased amounts of wall-associated extensins, and increased susceptibility to *Botrytis cinerea*, compared with wild type. Transgenic *Brachypodium* showed reductions in monomeric and dimeric ferulic acids and increased susceptibility to *Bipolaris sorokiniana.* Upon infection, transgenic *Arabidopsis* and *Brachypodium* plants also showed increased expression of several defense-related genes compared with wild type. These results demonstrate a role, in both monocot and dicot plants, of polysaccharide feruloylation in plant CWI, which contributes to plant resistance to necrotrophic pathogens.

## Introduction

In the heterogeneous, complex, and highly dynamic plant cell wall, structural polysaccharides form a cross-linked macromolecular network consisting of cellulose, hemicellulose, pectin, glycoproteins, and (in some specialized tissues) lignin (Albersheim et al., 2010). Cell wall constituents function in regulation of plant growth, development, signal transduction, and responses to environmental stresses (Somerville et al., 2004; Lionetti et al., 2012; Lionetti and Metraux, 2014; Tenhaken, 2015). In addition, plant biomass represents a valuable source of feed, fiber, and fuels, including a potential alternative to petroleum-based fuels. Cell wall lignification and the crosslinking of cell wall polysaccharides (Hatfield et al., 1999) hampers cell wall breakdown to simple sugars that can be used as an energy source by animals or be fermented to bioethanol (Himmel et al., 2007).

As plants are sessile, they have evolved a complex defense network to combat stresses, both biotic and abiotic, to survive in their environments. Many signaling pathways involved in plant stress responses and innate immunity have been identified and characterized (Jones and Dangl, 2006). Recent work has identified cell wall-related defense mechanisms, termed cell wall integrity (CWI) control (Ringli, 2010; Seifert and Blaukopf, 2010; Hamann and Denness, 2011; Pogorelko et al., 2013a; Bellincampi et al., 2014). As the first line of defense for plants, the cell wall must be rigid enough to maintain cell turgor and prevent pathogen invasion, but accommodate cell expansion, division, and organogenesis. Hence, the cell wall must respond rapidly to environmental changes without compromising its essential functions. Different cell wall components function in controlling cell expansion (Wolf et al., 2012) and defense responses (Nicaise et al., 2009; Tenhaken, 2015).

Certain phenolic compounds have key effects on cell wall structure, recalcitrance to degradation, and functions in defense (Buanafina et al., 2008; Pogorelko et al., 2011; Lionetti et al., 2015). For example, hydroxycinnamic acids, such as ferulic, sinapic, and coumaric acids, have been implicated in structural integrity, and some participate in crosslinking of cell wall constituents (Grabber et al., 1998), due to their ability to form homodimers, either photo-induced (Ralph et al., 1994) or peroxidase-assisted (Hartley et al., 1990). In particular, ferulate crosslinking has been broadly investigated, especially in monocot species (Ishii, 1997; Buanafina, 2009). As the predominant hydroxycinnamic component of grass cell walls, ferulate dimers crosslink hemicellulosic polymers via an ester linkage to the arabinose side chain of arabinoxylan or can be ether-linked to lignin (Ralph et al., 1995; Hatfield et al., 1999). In some dicots, by contrast, ferulic acid has been implicated in the crosslinking of pectic arabinans and galactans (Fry, 1982; Caffall and Mohnen, 2009). It has also been suggested that ferulic acid may crosslink pectins with wall-bound extensins, to negatively regulate cell expansion (Qi et al., 1995).

Ferulate can form homo- and heterodimers in numerous ways, such as 8-O-4, 8-8, and 4-O-5 linkages and ferulates can also bind in 8-5 and 5-5 conformations (Ralph et al., 2004). These dimers are considered an important structural aspect of the cell wall, but also hinder degradation for industrial purposes, such as biofuel production. Evidence indicates that ferulic acid (FA) affects plant-pathogen interactions, and that phenolic compounds are often induced in response to biotic stresses. FA is thought to play a role in fungal resistance and to be an important insect deterrent (Santiago and Malvar, 2010; Buanafina and Fescemyer, 2012). Apple leaves inoculated with the apple scab fungus *Venturia inaequalis* showed an increase in overall phenolic content, including FA (Mikulic Petkovšek et al., 2008). A negative correlation between FA concentration and disease severity was observed in maize exposed to *Fusarium graminearum* (Bily et al., 2003). Moreover, susceptibility to insect pathogens was negatively correlated with hydroxycinnamic acid content in maize (Bergvinson et al., 1994; García-Lara et al., 2004). Thus, these phenolic compounds may have key roles in cell wall structure and disease resistance.

Pathogens have evolved enzymatic mechanisms to target cell wall structure, including phenolic compounds; these enzymes have potential uses for improving digestibility of biomass. For example, some fungal species, such as *Aspergillus nidulans*, produce ferulic acid esterase (FAE), which hydrolyzes ester linkages between FA and cell wall polysaccharides of the host. Several studies investigated the effects of the ectopic expression of fungal FAE on plant cell wall composition and phenolic function. Vacuolar-targeted expression of a fungal FAE in *Lolium multiflorum* induced auto-digestion of hemicellulose after cell death (Buanafina et al., 2006). Targeted expression of *Aspergillus niger* FAE to the vacuole (Buanafina et al., 2008) or to the apoplast, endoplasmic reticulum, and Golgi (Buanafina et al., 2010) caused a reduction of cell wall feruloylation in *Festuca arundinacea* with a consequent improvement in cell wall digestibility. *Aspergillus* FAE, when added to fungal xylanase, improves the release of reducing sugars in oat hulls and in wheat bran (Faulds and Williamson, 1995; Yu et al., 2003). Arabinan-bound ferulic acid has been implicated in regulation of stomatal aperture of *Commelina communis*, and may confer flexibility in guard cells by preventing homogalacturonan (HGA) from binding calcium (Jones et al., 2003). Treatment with FAE, which hydrolyzes crosslinks between arabinans, allows HGA domains to associate through calcium crosslinked chain packing, thus reducing flexibility of the guard cell walls (Jones et al., 2003).

*Arabidopsis* plants expressing feruloyl esterase from *A. nidulans* have been previously generated and demonstrated to have reduced cell wall feruloylation and increased enzymatic saccharification of acid-pretreated plant biomass (Pogorelko et al., 2011). In this study, transgenic *Brachypodium* plants expressing the same microbial esterase were generated and characterized. Cell wall modifications in *Arabidopsis* and *Brachypodium* transgenic plants caused by expressed feruloyl esterase were characterized. The effect of these cell wall modifications on plant susceptibility to the necrotrophic fungi *B. cinerea* and *B. sorokiniana* was investigated and the results provide new insights into the relationship between the cell wall feruloylation, cell wall accessibility to degrading enzymes, and plant resistance to biotic stress. This study thus demonstrated that a reduction in cell wall cross-linking compromises plant resistance to pathogens.

## Materials and Methods

### Plant Growth Conditions

*Arabidopsis* seeds were planted in wet LC-1 potting soil mix (Sun Gro Horticulture, Agawam, MA, USA) and plants were grown in a growth chamber with controlled conditions: 16-h light/ 8-h dark at 21°C, with relative humidity of 65% and light intensity of 160 μmol s^-1^ m^-2^.

For the extensin quantification experiments, *Arabidopsis* seedlings were grown on plates in the same growth chamber as above. Seeds were sterilized with sequential treatments of 70% ethanol and 0.5% bleach, washed multiple times with sterile water and planted on -strength Murashige and Skoog medium (Murashige and Skoog, 1962) with 2% sucrose and 0.3% Gelrite (Research Products International, Mt. Prospect, IL, USA).

*Brachypodium* plants were grown in the same conditions as *Arabidopsis*. After a vernalization period of 14 days in the dark at 4°C to maximize germination rate, pots were transferred to a growth chamber maintained at 21°C, 65% relative humidity, and 160 μmol s^-1^ m^-2^ light intensity.

### Transformation of *Brachypodium* Plants with *AnFAE*

*Brachypodium* transgenic plants were prepared as described previously (Pogorelko et al., 2013b). The *A. nidulans AnFAE* cDNA (AN5267.2) was amplified from *Pichia pastoris* recombinant strains (Bauer et al., 2006), which were obtained from the Fungal Genetics Stock Center^1^. The cloned *AnFAE* sequence was amplified by PCR with primers AnFAE-F and AnFAE-R containing restriction sites *KpnI* and *HindIII*, respectively. After restriction digest of *AnFAE*, the fragment was ligated into a cassette containing (5′ to 3′) sequences encoding: a *Zea mays* β-expansin signal peptide, AnFAE, and a green fluorescent marker (smGFP), fused to the C-terminus of AnFAE. This cassette was then ligated into a pMLBart binary vector backbone, as described in Fursova et al. (2012). *A. tumefaciens*-mediated transformation of *Brachypodium* callus was performed by the Plant Transformation Facility at Iowa State University^2^. Diagrams of the transgenic expression cassettes for *Arabidopsis* and *Brachypodium* plants are shown in Supplementary Figure S1.

### Preparation of Apoplastic Fluid

Apoplast fluids were extracted from 6-week-old *AnFAE* and wild-type plants as described by Pogorelko et al. (2011). Briefly, aerial parts of the plants (0.5 g) were cut into 3–6 mm segments and placed vertically into a 10 mL syringe sealed with parafilm. Five mL of pre-cooled buffer (25 mM Tris-HCl, 50 mM EDTA, 150 mM Mg_2_Cl_2_, pH 7.4) was added and the syringe was placed under vacuum. After vacuum infiltration, the buffer was drained and the syringe was placed into a 15 mL centrifuge tube and centrifuged at 1000 g for 10 min. Apoplast fluid was collected from the bottom of the tube, transferred to a new tube, and frozen at -20°C.

### Feruloyl Esterase Activity Assay

For activity assays, apoplastic total protein was quantified using Bradford reagent (Bio-Rad Laboratories, Hercules, CA, USA). Then, equal amounts of protein per sample were incubated with 2 mM methyl ferulate (Sigma–Aldrich, St. Louis, MO, USA) in sodium phosphate buffer (pH 7.4) for 24 h. The products of reaction were extracted with ethyl acetate, evaporated, dissolved in 100% methanol, and analyzed by reverse-phase HPLC on a Prevail C18 5 μ column (4.6 mm × 50 mm; Grace Davison Discovery Sciences, Deerfield, IL, USA) with UV detection at 290 and 320 nm. Product and substrate were separated using a gradient of 0.1% trifluoroacetic acid in water (pH 2.8) and acetonitrile at 1 mL min^-1^ under following conditions: 0–3 min—95% water; 3–10 min—85% water; 10–20 min—70% water; 20–25 min—5% water; 25–30 min—95% water.

### Cell Wall Extraction

Cell walls were isolated from plants grown on soil as described in Zabotina et al. (2008). Whole aerial parts of plants were harvested and cut into 1-cm length segments. Tissue was frozen in liquid N_2_ and ground into a fine powder with a mortar and pestle. After homogenization, tissues were incubated in 80% ethanol at 80°C two times for 1 h, and further homogenized with a PolyTron (Kinematica, Inc., Bohemia, NY, USA) at 15,000 rpm for 5 min. The pellet was collected by centrifugation at 12,000 g and washed with 80% ethanol followed by several washes with 100% acetone until supernatant turned clear. The pellet was incubated in a solution of 20% SDS with 5 mM sodium metabisulfite at 4°C for 16 h and washed five times with distilled water. Finally, the pellet was incubated in 1:1 chloroform:methanol solution at room temperature for 20 min, washed three times with 100% acetone, and air-dried at 50°C.

### Analysis of Cell Wall Phenolic Acids

Phenolic acids and other hydroxycinnamates were extracted from prepared cell walls and analyzed as described by Pogorelko et al. (2011). Briefly, each total cell wall sample was weighed, incubated twice for 24 h in 2 ml 2M NaOH, and supernatants were combined. The supernatant mixture was acidified and phenolics were extracted with ethyl acetate, which was then evaporated with a stream of N_2_. Phenolics were then dissolved in 100% methanol and analyzed by reverse-phase HPLC on a Prevail C18 5 μ column (4.6 mm × 250 mm; Grace Davison Discovery Sciences, Deerfield, IL, USA) with UV detection at 290 and 320 nm. Phenolic acids were separated using a gradient of 0.1% trifluoroacetic acid in water (pH 2.8) and acetonitrile at 1 mL min^-1^ under following conditions: 0–10 min—95% water; 10–30 min—85% water; 30–40 min—70% water; 40–47 min—5% water; 47–55 min—95% water. To determine response factors, standard curves were created using mixtures of standard *p*-coumaric acid and ferulic acid, (all from Sigma–Aldrich, St. Louis, MO, USA) at different concentrations.

### Analysis of Cell Wall Sugars

To determine monosaccharide composition, 1 mg of dry, de-starched cell wall was hydrolyzed with 2 N trifluoroacetic acid at 120°C for 2 h. The hydrolysates were dried at 50°C, re-dissolved in water, and analyzed by high-performance anion-exchange chromatography with pulsed-amperometric detection using a CarboPac PA-20 column (3 mm× 150 mm; Dionex, Sunnyvale, CA, USA) as described earlier (Zabotina et al., 2008). Monosaccharides were separated using a gradient of 100mM NaOH in water at 0.5 mL min^-1^ under following conditions: 0–0.05 min—12 mM NaOH; 0.05–26 min—0.65 mM NaOH; 26–46 min—300 mM NaOH; 46–55 min—12 mM NaOH. Monosaccharide standards included L-Fuc, L-Rha, L-Ara, D-Gal, D-Glc, D-Xyl, D Man, D-GalA, and D-GlcA (all from Sigma–Aldrich, St. Louis, MO, USA). To determine response factors, standard curves were created using mixtures of all standard monosaccharides at different concentrations.

Reducing sugars were measured using the PAHBAH assay (Lever, 1972) with minor modifications. Briefly, 15 μL of supernatant was mixed with 135 mL of freshly prepared PAHBAH reagent (1 volume of 5% *p*-hydroxybenzoic acid hydrazide in 5% HCl mixed with 9 volumes of 1.25% trisodium citrate, 0.11% calcium chloride, and 2% sodium hydroxide) and heated at 95°C for exactly 6 min. Absorbance was measured at 410 nm using a microplate reader (BioTek Instruments, Inc., Winooski, VT, USA). Calculations were done using a standard curve prepared using different concentrations of Glc.

### Cell Wall Extensin Extraction and Quantification

Wild-type and transgenic *Arabidopsis* cell wall samples from 2-week-old whole plants (20–35 mg) were deglycosylated with anhydrous hydrogen fluoride (HF), as described previously (Sanger and Lamport, 1983). HF reactions were quenched, dialyzed (3.5 kDa molecular weight cut-off) against distilled deionized water, and then lyophilized. Lyophilized samples were re-suspended in water and fractionated into water-soluble (HF-soluble) and water-insoluble (HF-insoluble) fractions by centrifugation. HF-insoluble pellets were washed with water (~10–15 mL) to further separate HF-soluble components. Wash supernatants were pooled with HF-soluble fractions. Both HF-soluble and HF-insoluble fractions were lyophilized and weight recoveries were recorded on a microbalance. Untreated cell wall, HF-soluble, and HF-insoluble fractions were assayed for hydroxyproline after acid hydrolysis (constant boiling 6N HCl, 110°C, 24 h), as described earlier (Kivirikko and Liesmaa, 2009). Sample quantities permitting, analyses were performed in triplicate and error bars represent standard deviation.

### Enzymatic Digestions of Cell Wall

Digestion of hemicelluloses was done using 10 mg of dry cell wall material incubated with a mixture of 50 units of endo-1,4-β-xylanase M6 (rumen microorganism; Megazyme International, Wicklow, Ireland) and 5 units of endo-1,4-β-xylanase M1 (*Trichoderma viride*; Megazyme) in a 0.3-mL total volume of sodium phosphate buffer (pH 6.0) for 24 h at 37°C. For the digestion of pectins, 10 mg of dry cell wall material was incubated with a mixture of 50 units of endo-polygalacturonase (Megazyme International, Wicklow, Ireland) and 15 units of PME (PROZOMIX LTD, Haltwhistle, UK) in a 0.3-mL total volume of sodium phosphate buffer (pH 6.0) for 24 h at 37°C.

Saccharification assays were performed as described in Pogorelko et al. (2011) with some modifications. Stems of 6-week-old plants were cut into pieces approximately 0.3 cm long (5 mg of fresh tissue) and incubated in 0.1 ml of citrate buffer (pH 4.9) containing 4 units of cellulase (from *Trichoderma reesei*, Sigma–Aldrich, C62730) and 1 unit of cellobiase (from *A. niger*, Sigma–Aldrich, C6105) on the shaker at 37°C. At each time point, the reaction was terminated by heating at 100°C for 15 min, supernatants were collected by centrifugation at 10,000 g, and the amount of reducing sugars released was analyzed by *p*-hydroxybenzoic acid hydrazide (PAHBAH) assay.

### Inoculation of *Arabidopsis* with *Botrytis cinerea* Conidia

*Botrytis cinerea* strain SF1 (Lionetti et al., 2007) was grown for 15 days on potato dextrose agar (PDA) at 39 g liter^-1^ at 23°C with a 12-h photoperiod before spore collection. The spores were harvested by washing the culture surface of the agar and suspended in 5 mL of sterile distilled water. Spore suspensions were filtered through glass wool to remove residual mycelia and the concentration was determined using a Thoma chamber. Conidia, at a concentration of 5 × 10^5^ conidia mL^-1^, were germinated in potato dextrose broth (PDB) at 24 g L^-1^ at room temperature for 3 h. Fully developed leaves were detached from four 6-week-old *Arabidopsis* plants (three leaves/plant), grown in a growth chamber maintained at 22°C and 70% relative humidity, with a 12 h/12 h light-dark photoperiod. The detached leaves were placed in square petri dishes with petioles embedded in 0.8% agar. Six droplets of spore suspension (5 μL each) were placed on the surface of each leaf. Mock inoculation was performed using PDB. Leaves were incubated at 24°C with a 12-h photoperiod and lesion diameter was measured 48 h post inoculation. Lesion sizes were measured using IMAGE-J software (Abràmoff et al., 2004).

### Infection of *Brachypodium* with *Bipolaris sorokiniana*

Macroconidia of *B. sorokiniana* strain DSMZ 62608 (kindly provided by Prof. F. Favaron, University of Padua) were produced by culturing the fungus on PDA before spore collection. The conidia were collected by washing the culture surface with 3 mL of sterile water, and conidia concentrations were estimated using a Thoma chamber. The infection of *Brachypodium* leaves was performed as described previously (Pogorelko et al., 2013b). Briefly, fully expanded leaves were detached from 60-d-old *Brachypodium* plants grown under a 16 h/8 h light/dark cycle in a climate-controlled chamber at 22°C with a relative humidity of 70%. The leaves were cut to 5-cm lengths and placed in square petri dishes containing 0.8% agar. Four droplets (10 μL) of conidia suspension (1 × 10^6^ conidia mL^-1^), with 0.05% Tween 20, were deposited onto each leaf at a distance of about 2 cm from each other. Mock inoculation was performed using sterile distilled water with 0.05% Tween 20. The plates were incubated at 22°C under a 16-h/8-h light/dark cycle. Disease symptoms were recorded at 48 h and lesion sizes were measured using ImageJ (Abràmoff et al., 2004).

### RNA Extraction, cDNA Synthesis, and Real-Time qPCR

Total RNA was extracted from the uninfected, infected, and mock-inoculated leaves of 6-week-old plants using the SV Total RNA Isolation kit (Promega Corp, Madison, WI, USA), and cDNA synthesis was performed with the SuperScript III First Strand Synthesis system (Invitrogen Corp, Carlsbad, CA, USA) following the manufacturer’s recommendations. The Maxima SYBR Green qPCR Master Mix (2X; Thermo Scientific, Waltham, MA, USA) with appropriate primers (Supplementary Table S1) and the CFX-96 Thermal cycler (Bio-Rad) were used to determine relative expression of the genes. Relative expression levels were calculated in comparison with an appropriate control gene (for primer sequences, see Supplementary Table S1) from wild-type plants, and the *ACTIN2* gene (*At3g18780*; for *Arabidopsis*) whose expression level was not affected, was used as reference gene. Relative expression for *Brachypodium* samples was calculated by comparison with *GAPDH*. Gene expression levels in transgenic plants are presented relative to the expression of the same gene detected in wild-type plants (for which gene expression was set to 1). The comparative threshold cycle method (Schmittgen and Livak, 2008) was used for determining differences between transcript copy numbers in wild-type and transgenic plants. For calculation of relative expression levels for *B. cinerea* genes, a fungal house-keeping gene *ACTIN* (for primer sequences, see Supplementary Table S1) was used as a reference gene.

### Chlorophyll Extraction and Quantification

Chlorophyll extraction was performed as described in Lolle et al. (1997). Whole rosette leaves were isolated from 4-week-old *Arabidopsis* plants, then carefully weighed and placed in a 24-well plate. Leaves were immersed in dimethyl sulfoxide at room temperature, covered in foil, and placed on a shaker for incubation. Aliquots of supernatant were taken from solution every 10 min for 80 min. Absorption spectra at 664 and 647 nm were then measured. Chlorophyll was quantified as micromoles per mg tissue using the equation: chlorophyll (micromoles) = 7.93(A_664_) + 19.53(A_647_).

### Peroxide Staining of *Arabidopsis* Leaves

*Arabidopsis* leaves were assayed for hydrogen peroxide accumulation according to the protocol outlined by Daudi and O’Brien (2012). An aqueous solution of 1 mg/mL 3,3′-diaminobenzidine (DAB)(Acros Organics, New Jersey, USA) was prepared and adjusted to pH 3.0 with HCl. Tween-20 and Na_2_HPO_4_ were added to the solution to final concentrations of 0.05% and 10 mM, respectively. Rosette leaves from different plants were harvested and placed in a 24-well plate, then immersed in DAB solution. Samples were then vacuum infiltrated twice to ensure DAB solution penetrated the tissues. The 24-well plate was covered and placed on a shaker for 24 h.

At the 24 h time-point, leaf samples were placed in a bleaching solution of 3:1:1 ethanol: acetic acid: glycerol and heated to 95°C for 15 min. After boiling, fresh bleaching solution replaced the boiled solution and leaves were stored at 4°C until ready for imaging.

## Results

### Generation and Characterization of Transgenic *Brachypodium* Plants Expressing *Aspergillus nidulans* Feruloyl Esterase

**FIGURE 1:**
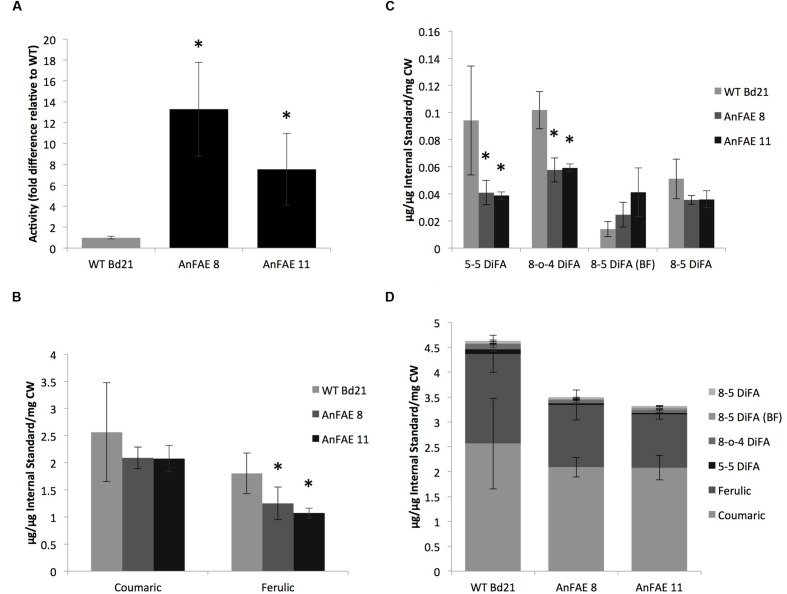
**Analysis of AnFAE activity and quantification of hydroxycinnamates in *Brachypodium* plants. (A)** Enzyme activity assay from *Brachypodium* apoplast. Quantification was done by measuring quantity and calculating the ratio of product (ferulic acid) to substrate (methyl ferulate). This ratio was then normalized to the average wild-type (WT) ratio to calculate fold difference. **(B,C)**, Quantification of hydroxycinnamate monomers and dimers in the *Brachypodium* cell wall. Compounds were identified under UV, then quantified based on peak area and normalized using an internal standard (cinnamic acid). Micrograms of each sample were determined using standard curves of known compounds. **(D)** Quantification of total hydroxycinnamates in *Brachypodium* cell wall. All peaks from quantitative analyses were added to determine overall reduction in total cell wall phenolics. Asterisks **(A–C)** indicate data sets significantly differently between AnFAE and wild-type plants, according to a Student’s *t*-test (*p* < 0.05).

To introduce *A. nidulans* feruloyl esterase (AnFAE), which we previously used to reduce the amount of ferulic acids in *Arabidopsis* plants (Pogorelko et al., 2011), to *Brachypodium* plants, a construct harboring *AnFAE* was prepared using the binary vector described by Fursova et al. (2012). Two selected independent transgenic *Brachypodium* lines were tested for production of AnFAE in the extracellular matrix by assaying enzymatic activity in their apoplastic fluids, with methyl ferulate as a substrate. All apoplastic fluids extracted from transgenic plants showed significantly higher feruloyl esterase activity in comparison with wild-type plants (**Figure 1A**).

To demonstrate that AnFAE expressed in *Brachypodium* apoplast reduces the amount of ferulic acids (FA), we quantified the total contents of FA, di-FA, and *p*-coumaric acid in cell walls extracted from aerial parts of transgenic and wild-type plants. Amount of both hydroxycinnamic acids present in cell wall extracted from transgenic plants varied among different plants; however, both lines had significantly less FA in comparison with wild-type plants (**Figure 1B**). In addition, cell walls from transgenic plants had lower amount of two types of di-FA (5–5 and 8-O-4 di-FA), whereas the content of two other di-FAs detected in *Brachypodium* cell wall (8–5 and 8–5 benzofuran di-FA) did not significantly change (**Figure 1C**). The total content of mono- and di-FA was also significantly lower in transgenic plants in comparison with wild-type plants (**Figure 1D**).

To investigate the effect of cell wall de-feruloylation on the polysaccharide composition, monosaccharide analyses of cell walls extracted from transgenic and wild-type plants were performed. For these analyses, *Brachypodium* transgenic lines AnFAE 8 and AnFAE 11, which showed strong reduction of FA in their cell walls, were also analyzed separately. A significant increase of xylose and reduction of glucose was observed in both transgenic lines in comparison with wild-type cell walls (**Table 1A**), indicating an increase in xylan content and a decrease in 1,3;1,4-mixed glucans in cell walls of *Brachypodium* transgenic plants, most likely in response to a reduction of polysaccharide cross-linking.

**Table 1:** Monosaccharide composition (mol %) of cell walls from wild-type (WT) and *AnFAE*-expressing *Brachypodium* and *Arabidopsis* plants.

(A)	Fuc	Rha	Ara	Gal	Glc	Xyl	GalA	GlcA	
WT Bd21	0.9 ± 0.3	0.6 ± 0.4	12.7 ± 2.8	3.4 ± 1.1	31.2 ± 9.5	49.6 ± 6.5	0.8 ± 0.7	0.7 ± 0.7	
AnFAE 8	0.7 ± 0.3	0.5 ± 0.6	14.8 ± 0.7	3.8 ± 0.1	**13.4 ± 1.1**	**64.3 ± 1.6**	1.9 ± 0.6	0.7 ± 0.8	
AnFAE 11	0.6 ± 0.3	0.7 ± 0.6	14.5 ± 1.1	3.5 ± 0.4	**13.1 ± 2.4**	**65.6 ± 2.9**	1.2 ± 0.5	0.7 ± 0.6	

**(B)**	**Fuc**	**Rha**	**Ara**	**Gal**	**Glc**	**Xyl**	**Man**	**GalA**	**GlcA**	

WT Col-0	1.2 ± 0.3	4.6 ± 0.4	5.3 ± 1.7	8.8 ± 2.5	9.0 ± 0.1	55.2 ± 3.2	6.9 ± 0.9	7.6 ± 1.7	1.5 ± 0.8
AnFAE	1.5 ± 0.5	5.3 ± 2.1	4.5 ± 1.2	8.7 ± 2.2	8.7 ± 0.4	56.0 ± 5.6	6.5 ± 0.3	7.1 ± 1.4	1.6 ± 0.6

***(A)** Cell wall composition of *Brachypodium* non-cellulosic polysaccharides from WT and *AnFAE* plants. **(B)** Cell wall composition of *Arabidopsis* non-cellulosic polysaccharides from WT and *AnFAE* plants. Numbers in bold represent statistical significance (Student’s *t*-test, *p* < 0.05, *n* = 3–6).*

In addition, monosaccharide analysis was performed for cell walls extracted from one previously characterized line of transgenic *Arabidopsis* plants expressing the same fungal enzyme (AnFAE). *Arabidopsis* transgenic plants showed a reduction in mono-FA content (~50%), but no di-FA was detected in their cell walls (Pogorelko et al., 2011). In contrast to *Brachypodium*, *Arabidopsis* transgenic plants did not display any alteration in monosaccharide composition (**Table 1B**).

### *AnFAE* in the Apoplast Affects *Arabidopsis* and *Brachypodium* Cell Wall Digestibility

**FIGURE 2:**
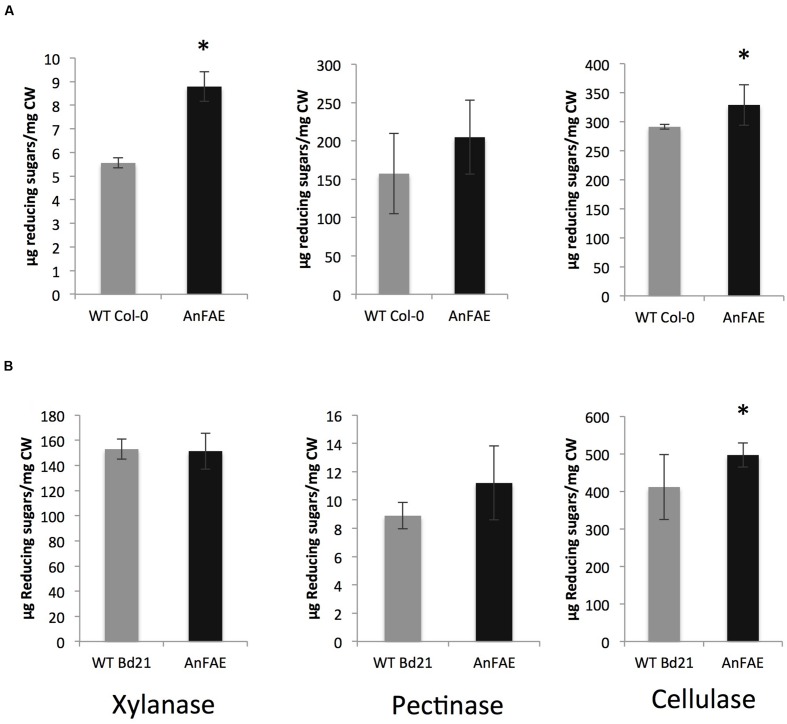
**Amount of reducing sugars released after incubation of cell wall material from 8-week old plants with wall-degrading enzymes. (A)** *Arabidopsis* cell wall treated with xylanase, pectinase, and cellulase. **(B)** *Brachypodium* cell wall treated with xylanase, pectinase, and cellulase. Asterisks indicate significant differences according to Student’s *t*-test (*p* < 0.05, *n* = 3–6).

To determine whether reduction of cell wall feruloylation alters cell wall digestibility, we measured the amount of reducing sugars released during treatment of cell walls extracted from *Arabidopsis* and *Brachypodium* transgenic and wild-type plants with either xylanase, pectin methyesterase plus polyglacturonase (PME+PG) or a cellulase cocktail. Treatment of cell walls from *Arabidopsis* transgenic plants with xylanases and cellulases released significantly more reducing sugars in comparison with wild-type cell wall, whereas amount of sugars released by PME+PG treatment was not different (**Figure 2A**). Cell walls extracted from *Brachypodium* transgenic plants released higher amount of reducing sugars only after treatment with cellulases, but not after xylanase or PME+PG treatment (**Figure 2B**).

### *AnFAE*-expressing Plants Have Higher Susceptibility to Necrotrophic Fungal Pathogens

**FIGURE 3:**
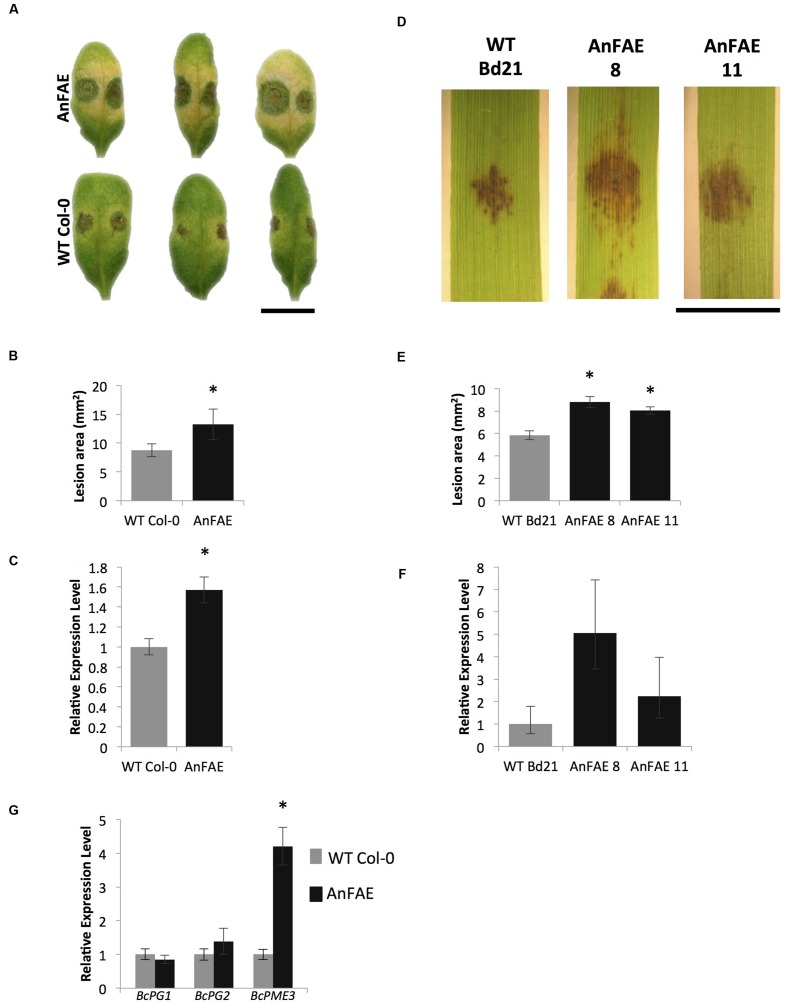
**Analysis of *Arabidopsis* and *Brachypodium* transgenic plants susceptibility to fungal necrotrophs. (A,D)**, *B. cinerea* and *B. sorokiniana* symptoms on *Arabidopsis* and *Brachypodium* transgenic AnFAE and wild-type leaves. **(B,E)**, Measurements of lesion areas on *Arabidopsis* and *Brachypodium* leaves 48 h post inoculation (HPI). **(C,F)**, Relative expression levels of *B. cinerea*-*Actin* in *Arabidopsis* and of *B. sorokiniana GAPDH* in *Brachypodium* AnFAE and wild-type leaves 48 HPI. **(G)** Expression of *B. cinerea* virulence-related genes in *Arabidopsis* AnFAE and Col-0 plants. Asterisks indicate statistical significance according to Student’s *t*-test (*p* < 0.05, *n* = 3). Scale bars = 1 cm.

Since modification of cell wall composition, and specifically reduction of FA, may affect plant-pathogen interactions (Venkata et al., 2013), we next used pathogen infection to challenge the transgenic *Arabidopsis* generated previously (Pogorelko et al., 2011) and the *Brachypodium* plants generated in this study. We measured the susceptibility of *Arabidopsis* plants expressing *AnFAE* and wild-type Col-0 plants by inoculating leaves with *B. cinerea* conidia and evaluating symptoms 48 h post inoculation (hpi). Similarly, leaves of two *Brachypodium* transgenic lines and wild-type plants were infected with *B. sorokiniana* and symptoms were evaluated at 48 hpi. Both *Arabidopsis* and *Brachypodium* transgenic plants showed a significant increase in the areas of lesions produced by the pathogens in transgenic plants compared with wild-type plants (**Figures 3A,B,D,E**).

In *Arabidopsis*, higher accumulation of *B. cinerea Actin* transcripts was detected in infected transgenic leaves in comparison with infected wild-type leaves. In *Brachypodium*, a slightly higher, though not statistically significant, amount of *B. sorokiniana GLYCERALDEHYDE-3-PHOSPHATE DEHYDROGENASE-LIKE* (*GADPH*) was detected in infected transgenic leaves in comparison with infected wild-type leaves (**Figures 3C,F**).

*Botrytis cinerea* expresses a multitude of wall-degrading enzymes during pathogenesis and we also measured transcript levels of *BcPG1*, *BcPG2*, and *BcPME3* during infection to investigate possible relationships between plant susceptibility, cell wall structural integrity, and fungal virulence. While the expression of *PG* genes did not change in transgenic plants, up-regulation of *BcPME3* was observed in infected *Arabidopsis* transgenic leaves (**Figure 3G**). These results indicate that the expression of *AnFAE* in *Arabidopsis* and *Brachypodium* can increase susceptibility to fungal necrotrophs.

### De-feruloylation of Cell Walls Alters the Expression of Pathogen-responsive Genes during Pathogenesis

Increased susceptibility to pathogens can be caused by the release and accumulation of ferulate in the apoplastic fluid of transgenic plants or by compromised CWI, which may induce plant-specific responses. Measurement of FA contents in apoplastic fluids of transgenic *Arabidopsis* plants did not reveal the presence of detectable free FA (Supplementary Figure S2).

**FIGURE 4:**
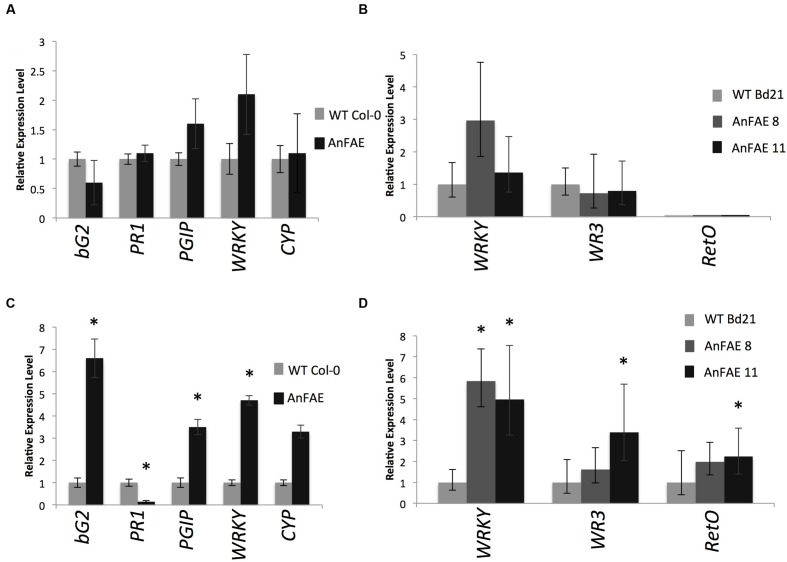
**Real-time qPCR analysis of selected pathogen-inducible defense genes in mock-infected (**A**-*Arabidopsis*, **B**-*Brachypodium*) and 48 HPI (**C**-*Arabidopsis*, **D**-*Brachypodium*) plants.** Gene expression levels in transgenic plants normalized to the expression of the same gene detected in wild-type plants (for which gene expression was set to 1), *ACTIN2* was used as reference gene for *Arabidopsis* and *GAPDH* for *Brachypodium* expression analysis. The comparative threshold cycle method was used for determining differences between transcript copy numbers in wild-type and transgenic plants. Data represent average obtained for three independent transgenic lines. Asterisks indicate significant differences between transgenic plants and wild-type plants (Student’s *t*-test, *P* < 0.05; *n* = 3).

To investigate whether the expression of fungal *AnFAE* and the consequent decrease of FA content in the cell wall affect the pathways involved in plant response to infection, we used RT-qPCR to measure the transcript levels of several known defense genes in infected and un-infected *Arabidopsis* and *Brachypodium* transgenic and wild-type plants. In healthy, un-infected transgenic plants, expression of these genes did not differ from their expression in wild-type plants (**Figures 4A,B**). However, several defense-related genes were significantly up-regulated in transgenic plants in comparison with wild-type plants upon infection (**Figures 4C,D**). Thus, in *Arabidopsis* transgenic plants, expression of *PGIP1*, *bG2*, and *WRKY40* was significantly higher than in wild-type plants, whereas the level of *PR1* transcript was significantly lower (**Figure 4C**). Some increase of *CYP81F2* was also observed in infected *Arabidopsis* plants but the difference was not statistically significant. In *Brachypodium*, expression of *WRKY40*, *WR3*, and *RetOX* was higher in comparison with infected wild-type plants (**Figure 4D**). A full list of genes assayed, including those not significantly different from wild-type and not amplified in *Brachypodium*, is included in Supplementary Figure S3.

### Reduction of Cell Wall Ferulic Acid Content Affects Extensin Extractability in Transgenic *Arabidopsis* Plants

**FIGURE 5:**
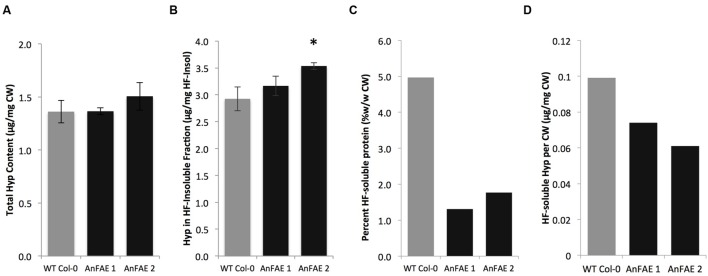
**Hydroxyproline content in cell walls of wild-type and two *AnFAE Arabidopsis* lines. (A)** Total hydroxyproline content in cell walls as determined by absorbance at 560 nm. **(B)** Hydroxyproline content in the HF-insoluble cell wall fraction (μg Hyp/mg HF-Insoluble). **(C)** Percent of HF-soluble protein per mg cell wall (mg/mg CW) **(D)** Hydroxyproline content in HF-soluble protein (μg Hyp/mg CW). Asterisks indicate statistical significance (Student’s *t*-test, *p* < 0.05, *n* = 3).

Previous work indicated that in dicots, FA may be involved in cross-linking pectins with extensins (Qi et al., 1995). Extensins are also induced during defense responses of *Arabidopsis* plants (Lamport et al., 2011). To investigate whether *Arabidopsis* plants expressing *AnFAE* have altered amounts of extensin in their cell walls, HF was used to deglycosylate cell walls. The HF-soluble fraction represents the pool of wall-associated extensins, while the HF-insoluble fraction represents the pool of crosslinked extensins (Mort and Lamport, 1977). The amount of hydroxyproline was quantified as an indicator of extensin contents. We found that the total extensin content was unchanged in transgenic and wild-type cell walls (**Figure 5A**). The amount of crosslinked extensin was slightly elevated in walls from *AnFAE* transgenic plants (**Figure 5B**). However, the proportion of HF-soluble protein per mg cell wall was significantly reduced in cell walls from *AnFAE* transgenic plants (**Figure 5C**). In the HF-soluble fraction, *AnFAE* cell wall samples contained lower amounts of hydroxyproline (**Figure 5D**). This indicates that reduction of cell wall feruloylation affects extensin self-cross-linking.

## Discussion

Increasing evidence demonstrates that changes in cell wall composition can stimulate plant responses similar to stress responses (Hamann, 2012; Pogorelko et al., 2013a). For example, *Arabidopsis* and tobacco plants expressing an attenuated version of endopolygalacturonase II from *A. niger* had improved resistance to fungal and bacterial pathogens and increased cell wall digestibility, but exhibited a dwarf phenotype (Capodicasa et al., 2004; Ferrari et al., 2007). Overexpression of a fruit-specific pectin methylesterase (PME) in wild strawberry induced defense-related gene expression and increased plant resistance to *B. cinerea* (Osorio et al., 2011). *Arabidopsis* and *Brachypodium* plants expressing either acetylxylan esterase or rhamnogalacturonan acetylesterase from *A. nidulans* exhibited higher resistance to fungal necrotrophs and had increased transcript levels for some defense-related genes (Pogorelko et al., 2013b). Expression of a thermostable xylanase in rice induced upregulation of plant defense-related genes such as *SOD*, *CAT*, and the xylanase inhibitor *RIXI* (Weng et al., 2013). Cell wall structures can also serve as a mechanical barrier to microbial penetration and thus influence plant resistance. Increased pectin methylesterification caused by overexpression of PME inhibitors (PMEI) decreased susceptibility to fungal and bacterial pathogens and viruses (Lionetti et al., 2007, 2012, 2014). *Arabidopsis* and wheat plants overexpressing *PMEI* had increased biomass and improved tissue saccharification (Lionetti et al., 2010; Volpi et al., 2011).

By contrast, modifications that compromise cell wall mechanical properties can potentially have the opposite effect, reducing plant resistance to pathogens and other stresses. For example, reduction in lignin content in soybean *Rpp2* plants was correlated with their loss of resistance to the soybean rust fungus *Phakopsora pachyrhizi* (Pandey et al., 2011). Therefore, such cell wall modifications require detailed characterization of plant fitness to be effective in applied crop biotechnology.

Earlier, we reported that *Arabidopsis* plants expressing the *A. nidulans* feruloyl esterase AnFAE exhibited significant reduction of cell wall feruloylation and increased biomass saccharification by fungal cellulases after acid pretreatment (Pogorelko et al., 2011). In this study, we additionally generated the *Brachypodium* transgenic plants expressing the same AnFAE protein and confirmed reduction of monomeric and dimeric FA in their cell walls, which increased *Brachypodium* cell wall degradability by fungal cellulases. Using the *Arabidopsis* transgenic plants created earlier and the *Brachypodium* transgenic plants created in this study, we investigated the effect of cell wall deferuloylation on plant susceptibility to fungal necrotrophic pathogens and obtained new insights into the potential role of FA in plant CWI.

The increased degradability of cell walls observed in the plants expressing *AnFAE* indicates that reduction of FA affects the accessibility of polysaccharides in both type I (dicots) and type II (grasses) cell walls. While the effect of monomeric and dimeric FA reduction on *Brachypodium* cell wall digestibility demonstrated in this study was not surprising and conforms to similar observations in other grasses (Buanafina et al., 2010; Harholt et al., 2010), to the best of our knowledge, there are no reports about the effect of FA on the digestibility of the cell wall in dicots. Our results indicate that most likely, in the *Arabidopsis* cell wall, FA is esterified to fatty acids in the cutin (Rautengarten et al., 2012; Fich et al., 2016) as well as to the polysaccharides in the cell wall. It is possible that monomeric FA forms cross-links between those molecules creating a connection between the cell wall and cutin, affecting the accessibility of cell wall degrading enzymes or the integrity of the cutin. However, these possible changes in cutin-cell wall connections do not seem to affect cutin permeability/integrity, since our experiments studying chemical leaching of chlorophyll showed no difference between *AnFAE* and wild-type plants (Supplementary Figure S4). It cannot be excluded that the reduction of FA occurs exclusively on polysaccharides within the cell wall.

In dicots, FA may cross-link polysaccharides with extensins, as previously proposed (Qi et al., 1995), in response to pathogen attack (Deepak et al., 2010). While in *AnFAE*-expressing plants, total extensin content remained unchanged in comparison with wild-type *Arabidopsis* plants (**Figure 5A**), the amount of loosely associated HF-soluble extensin significantly decreased (**Figure 5D**) due to the increased proportion of self-cross-linked extensin that became insoluble. This shift from loosely associated extensins, toward self-cross-linked extensins may occur in response to reduction of polysaccharide-extensin interconnections. It is plausible to propose that crossed-linked extensins contribute to recalcitrance of the type I cell wall in dicots somewhat similarly to diFA in monocot type II cell walls. Hence, the reduction of FA content in transgenic *Arabidopsis* plants expressing *AnFAE* can induce higher self-cross-linking of extensins as a compensatory response by the plants to fortify their cell walls.

Reduction of cell wall crosslinking and increase of their degradability in *AnFAE* plants negatively affects the plant resistance to necrotrophic fungal pathogens. Both the *Arabidopsis* and *Brachypodium* transgenic plants showed significantly higher susceptibility to *B. cinerea* and *B. sorokiniana*, respectively, consistent with higher digestibility of their cell walls. It is plausible to expect that mechanical strength of the wall with reduced di-FA cross-linking in *Brachypodium* plants is compromised, which makes it more accessible to the cell wall degrading enzymes secreted by fungus. Most likely, in *Arabidopsis* transgenic plants, the strength of cell walls is reduced as well, though perhaps due to reduction of polysaccharide-extensin connections. This notion will need further investigation in the future.

Transcript analysis of the known plant defense-related genes *PR1*, *bG2*, *PR5*, *PAD3*, *WR3*, *JR1*, *WRKY40*, *PGIP1*, *CYP81F2*, and *RetOx* (Galletti et al., 2008; Raiola et al., 2011), as well as susceptibility genes *PME3* (Raiola et al., 2011), *PMR4* (Nishimura and Somerville, 2002), *PMR5* (Vogel et al., 2004), *PMR6* (Vogel et al., 2002), and *MYB46* (Ramírez et al., 2011), demonstrated that none of the investigated genes were constitutively up- or down-regulated in *AnFAE* plants. Also, peroxide staining did not show an alteration in the accumulation of hydrogen peroxide in the leaves of transgenic plants with respect to wild-type plants. (Supplementary Figure S5). These results indicate that a perturbation of FA-mediated crosslinking in the cell wall does not induce defense responses in the absence of biotic stress, in contrast to what was observed in the case of post-synthetic cell wall de-acetylation (Pogorelko et al., 2013b) or pectin modification (Ferrari et al., 2008). However, *Arabidopsis* plants expressing *AnFAE* showed higher expression of *bG2* (*PR2*), *PGIP1*, and *WRKY40*, and repression of *PR1* upon *B. cinerea* infection in comparison with wild-type plants. By contrast, *Brachypodium* plants expressing *AnFAE* showed higher expression of genes homologous to *Arabidopsis WRKY40*, *WR3*, and *RetOx*, in comparison with wild-type plants, upon infection with *B. sorokiniana*. The pattern of affected defense-related genes in *AnFAE* plants was somewhat different from those that were differentially expressed in the plants with reduced cell wall acetylation or reduced homogalacturonan contents (Ferrari et al., 2008; Pogorelko et al., 2013b). This could suggest that different alterations of cell wall structure initiate different CWI signaling pathways, which activate discrete defense pathways in response to pathogen infection.

The *bG2* gene encodes a β-glucanase, which is up-regulated during the *Arabidopsis* response to biotic stress (Dong et al., 1991). This gene was also constitutively up-regulated in *Arabidopsis* plants expressing rhamnogalacturonan acetylesterase in response to reduced acetylation of pectin and xyloglucan. Moreover, basal β-1,3-glucanase activity was significantly higher in tobacco and *Arabidopsis* lines expressing high levels of *A. nidulans* polygalacturonase (Ferrari et al., 2008). This indicates that despite the presence of specific responses, the activation of β-glucanase is a common response to the loss of CWI, regardless of the polysaccharide type in the cell wall being perturbed.

The accumulation of the cell wall associated polygalacturonase inhibiting proteins (PGIPs) is considered to contribute to resistance against polygalacturonase-producing pathogenic fungi (De Lorenzo et al., 2001). A higher level of *PGIP* expression was observed in *Arabidopsis AnFAE*-expressing plants in response to fungal treatment. It is possible that although *AnFAE* plants try to defend themselves, the integrity of their cell walls is altered to the extent that it cannot be compensated by the activation of these defense genes.

The *Arabidopsis WOUND RESPONSIVE 3* (*WR3*) encodes a high-affinity nitrate transporter, which was proposed to be involved in jasmonic acid-independent wound signal transduction (Titarenko et al., 1997) and was shown to be activated by treatment with fungal-derived oligosaccharide elicitor (Rojo et al., 1999). WR3 was proposed to be involved in hypersensitive-like cell death induction (Kim et al., 2008). Higher expression of the homologous gene in *Brachypodium AnFAE*-expressing plants upon infection might suggest that reduction of FA-mediated cross-linking in their cell walls increases the response of the plant upon wounding associated with the action of necrotrophic microorganisms.

After fungal inoculation, *Arabidopsis AnFAE*-expressing plants showed lower expression of the salicylic acid (SA)-dependent *PR1* gene, which was consistent with upregulation of the transcription factor gene *WRKY40*, considered to be a repressor of SA signaling (Xu et al., 2006; Zheng et al., 2006). This suggests that the weakening of cell walls due to reduction of FA-mediated crosslinks might affect either the SA metabolic pathway or activation of SA-dependent pathways. We did not observe higher accumulation of FA or any other phenolic acids in the apoplast of *AnFAE* plants in comparison with wild-type plants; therefore, we can eliminate the possibility that increased free FA in the apoplast contributes to repression of *PR1*.

The *B. cinerea* genome contains at least six endopolygalacturonase genes (*BcPGs*) (Wubben et al., 1999), of which *BcPG1* and *BcPG2* are required for virulence (Kars et al., 2005). At the same time, most pectinolytic enzymes cannot degrade highly methylated pectin. Therefore, the action of pectin methylesterase (PME), which de-methylesterifies pectin to pectate, is required. Although neither BcPME1 nor BcPME2 are required for fungal virulence, a possible involvement of BcPME3 was not excluded (Kars et al., 2005). Thus, we measured expression of fungal *BcPG1*, *BcPG2*, and *BcPME3* during infection to investigate possible relations between plant susceptibility, cell wall structural integrity and fungal virulence. Since different sets of cell wall degrading enzymes are secreted at different stages of fungal infection (Laluk and Mengiste, 2010), the higher expression of *BcPME3* but not of *BcPG1* and *BcPG2* during growth of *B. cinerea* on *AnFAE* plants, in comparison with wild-type plants might reflect the faster development of fungal colonies at the stage that requires contribution of *BcPME3*.

Based on the results of this study, we propose that some improvements of feedstock biomass digestibility can negatively affect plant stress resistance, so a better understanding of this trade-off can help in finding optimal directions in plant biotechnology.

## Conclusion

In this study, we demonstrated that alteration of plant cell wall feruloylation has a negative effect on plant fitness. While improving cell wall digestibility, reduction of ferulic acid content negatively affects plant resistance to biotic stress, most likely due to compromised CWI. Thus, in *Brachypodium*, reduction of FA-mediated cross-linking reduces cell wall strength, which leads to lower resistance against microbial penetration. Similarly, the cell wall in *Arabidopsis* becomes weaker, most likely due to changes in the polysaccharide-extensin network, but further detailed analyses are necessary to confirm this.

## Author Contributions

NR designed and conducted experiments for cell wall and expression analysis, enzyme activity assays, and wrote the paper. GP designed and conducted experiments for cell wall and expression analysis, and wrote the paper. VL designed experiments and conducted experiments in pathogen response. LC assisted with conducting experiments for cell wall analysis. MH designed and conducted experiments for extensin analysis. DB designed experiments for pathogen response. OAZ designed experiments, supervised all activity, and wrote the paper. All authors conducted revisions of the paper.

## Conflict of Interest Statement

The authors declare that the research was conducted in the absence of any commercial or financial relationships that could be construed as a potential conflict of interest.

## Footnotes

1 http://www.fgsc.net

2 http://www.agron.iastate.edu/ptf
