# Supplementary material for: Decreased Polysaccharide Feruloylation Compromises Plant Cell Wall Integrity and Increases Susceptibility to Necrotrophic Fungal Pathogens
Source: Front Plant Sci. 2016 May 10;7:630. doi: 10.3389/fpls.2016.00630 (PMC4862258; doi:10.3389/fpls.2016.00630)
Supplement: Supplementary file 1 [file Data_Sheet_1.pdf]

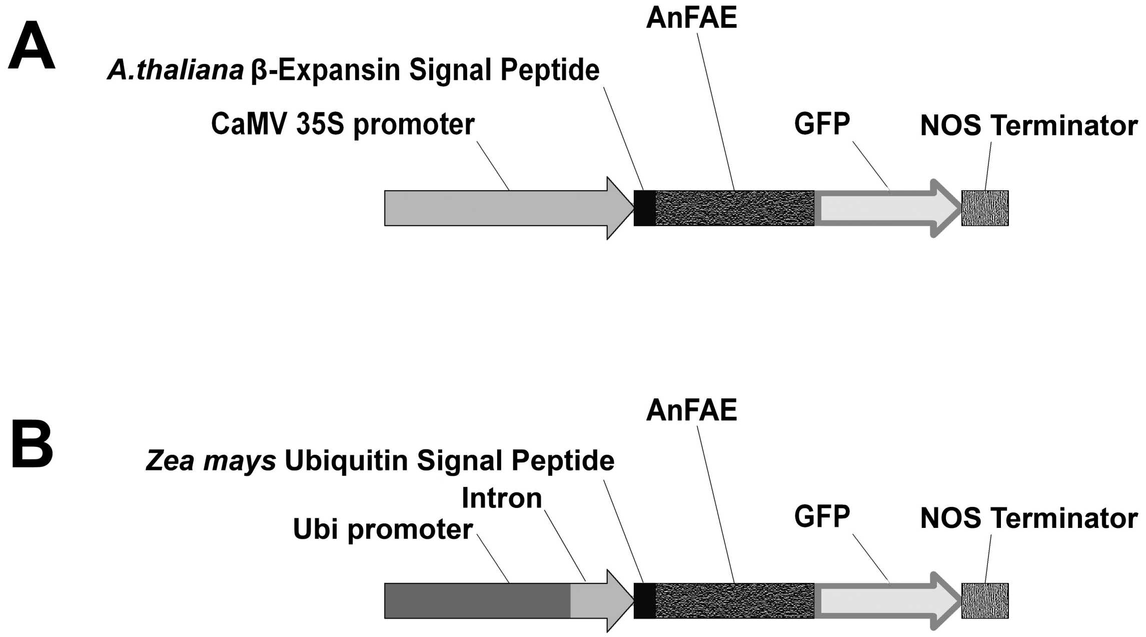

Supplementary Figure S1. Schematic diagram of expression cassettes used to express the genes in Arabidopsis (A) and Brachypodium (B) plants. Each cassette contains AnFAE from *Aspergillus nidulans* (AN5267.2) fused at the 3' end to smGFP (GFP), followed by a nopaline synthase (NOS) transcription terminator. Constructs used for transformation of Arabidopsis (A) contained four sequential CaMV 35S promoter regions for plant overexpression. To export this protein to the apoplast, a β-expansin signal peptide was fused to the 5' end of AnFAE. Constructs for Brachypodium transformation (B) contained the *Zea mays* ubiquitin gene promoter and its downstream intron (known to be required for suitable transcription). *Zea mays* ubiquitin signal peptide fused to the 5' end of AnFAE was used for export of protein to the apoplast.

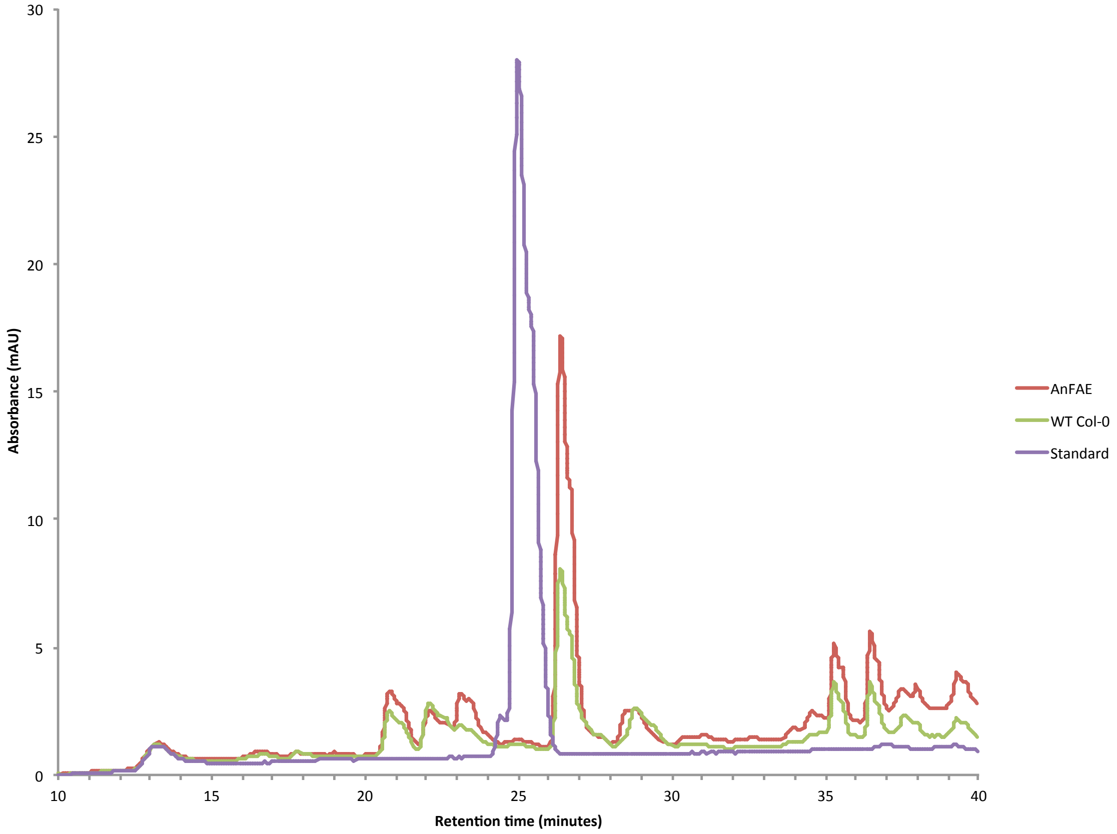

Supplementary Figure S2. Analysis of the apoplast fluids from *AnFAE* (red) and wild-type Col-0 (green) plants using reverse-phase HPLC on a Prevail C18 5µ column (4.6 x 250 mm; Grace Davison Discovery Sciences, Deerfield, IL, USA) with UV detection at 290 and 320 nm. Comparison of all obtained peaks with the chromatogram of the standard ferulic acid (purple) confirmed that plant apoplasts do not contain free ferulic acid.

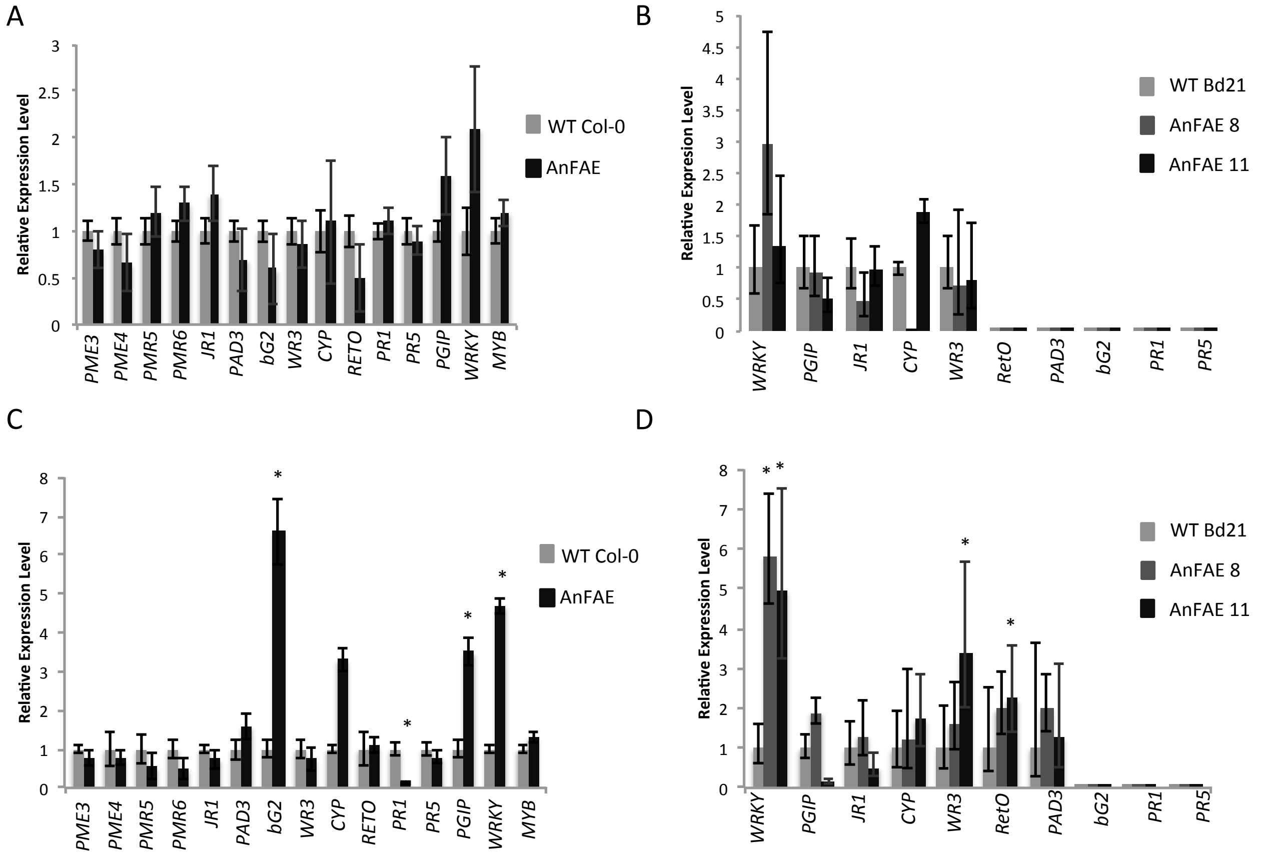

Supplementary Figure S3. Real-time qPCR analysis for all genes used in this study. Pathogen-inducible defense genes in mock-treated plants (A-Arabidopsis, B-Brachypodium) and in infected plants 48 HPI (C-Arabidopsis, D-Brachypodium). Transcript levels in transgenic plants normalized to the expression of the same gene detected in wild-type plants (for which gene expression was set to 1), *ACTIN2* was used as the reference gene for Arabidopsis and *GAPDH* was used for Brachypodium. The comparative threshold cycle method was used for determining differences between transcript copy numbers in wild-type and transgenic plants. Data represent average obtained for three independent transgenic lines. Asterisks indicate significant differences between transgenic plants and wild-type plants (Student's *t*-test, $P < 0.05$; $n = 3$).

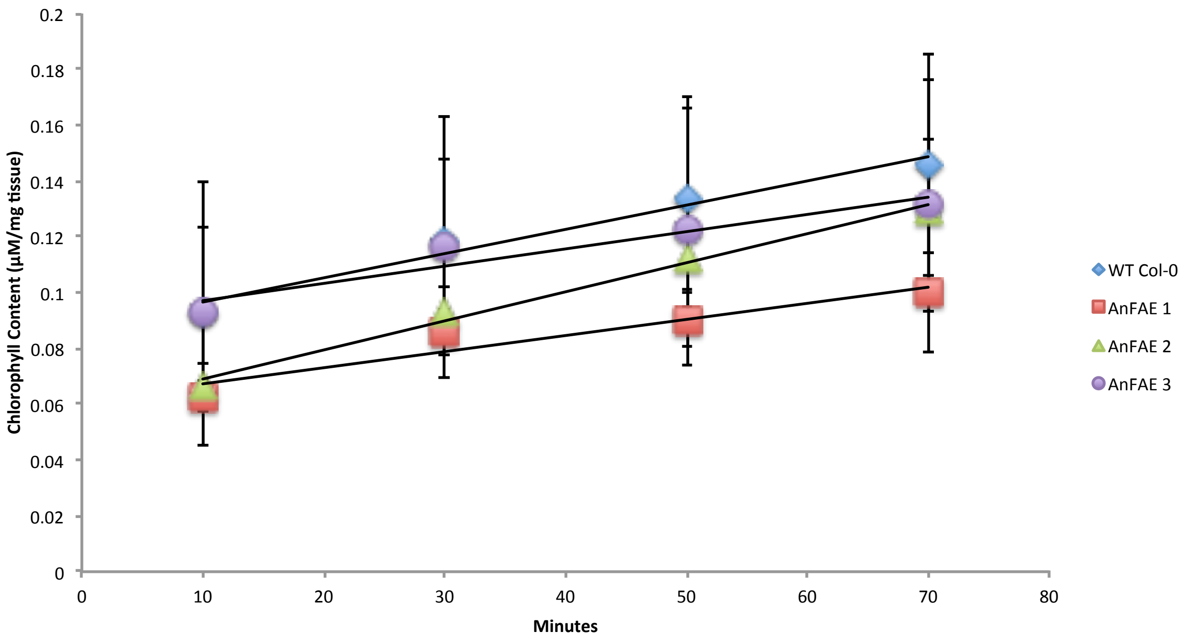

Supplementary Figure S4: Time-course of chlorophyll leaching from Arabidopsis leaves measurement using dimethyl sulfoxide with 10 minutes intervals. AnFAE 1, 2, and 3 represent three independent Arabidopsis lines expressing AnFAE. Error bars represent standard deviation. Samples were analyzed with student's t-test and no samples were found to be significantly different from WT Col-0.

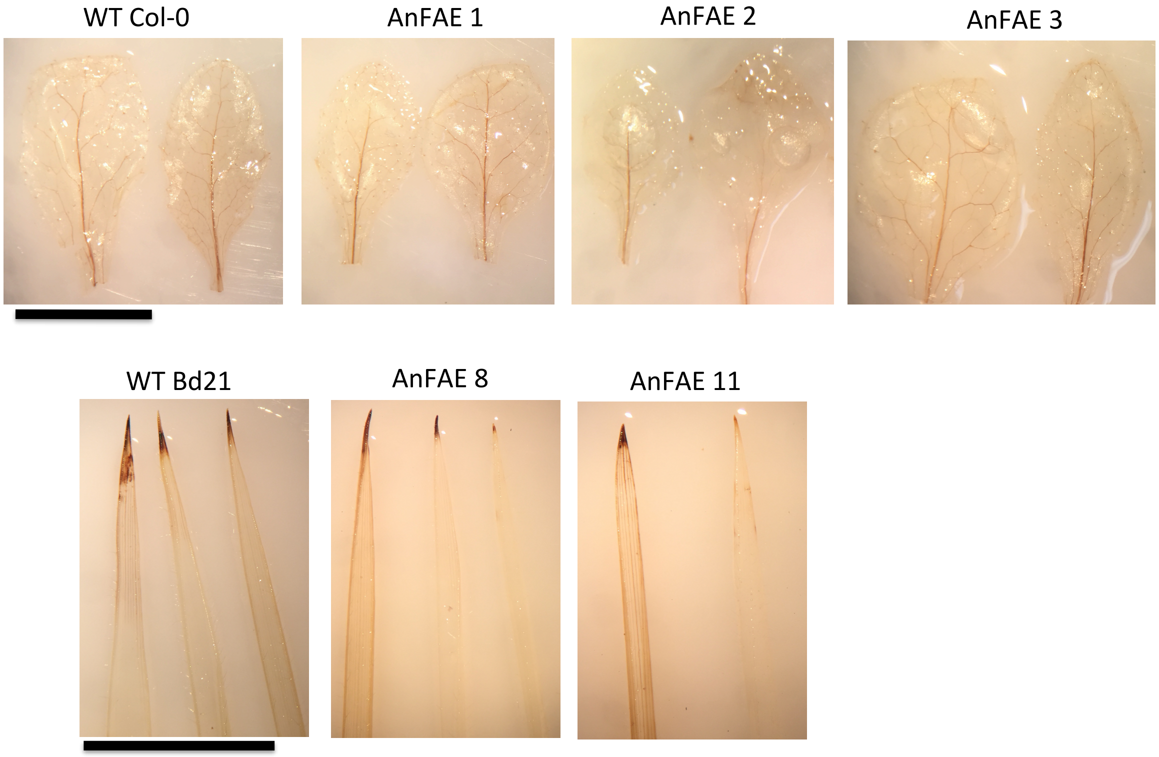

Supplementary Figure S5. Peroxide accumulation in Arabidopsis and Brachypodium leaves by 3,3'-diaminobenzidine (DAB) incubation for 24 hours. Arabidopsis AnFAE 1, 2, and 3 represent three independent Arabidopsis lines expressing AnFAE, compared to wild type Col-0. Two Brachypodium lines, AnFAE 8 and AnFAE 11, were compared to wild type Bd21. Scale bars = 1 cm.

Supplementary Table S1. Primer sequences used in this work (5’-3’):
AtPR1-F: TCTAAGGGTTCACAACCAGG
AtPR1-R: CCTTCTCGCTAACCCACATG
AtPR5-F: GAGGATCGGGAGATTGCAAA
AtPR5-R: GTCAGGGCAAGCGTTCTTGA
AtbG2-F: GACGCAAATCTCGACTCGGT
AtbG2-R: TCTCTATAGCTTTCCCTGGC
AtPAD3-F: ACTCTGGGAAAACGCAGATG
AtPAD3-R: CTTTGGCTTCCTCCTGCTTC
AtJR1-F: GTGTCGGGCTACTATGACAA
AtJR1-R: GGGCGCAACATTGACTCCAA
AtWR3-F: TTCGTGCCTACGCGGTTGAT
AtWR3-R: CTATCTTGGCCTTCCTCTTC
AtPGIP-F: CAGCTCAAGAATCTCGAGTT
AtPGIP-R: TCGATCCGGTTAAAGTCGAT
AtWRKY-F: CTAGAGACAATCCATCTCCA
AtWRKY-R: TGCTGCAACGGGTGTTGAAG
AtCYP-F: CAGCTGCACCACTTCTTGTT
AtCYP-R: CACCAGGACACGTTCTTCGT
AtRetO-F: AATGATGGATCGGATTCCGT
AtRetO-R: ACCGCTTGGATTGCTTCCAA
AtActin2_F: GAAACCCTCGTAGATTGGCA
AtActin2_R: CTCTCCCGCTATGTATGTCGC
AtPME3_F: TTCAGGTGTGAAAGCTGACG
AtPME3_R: GTCCGACCATCTCCCATAAA
AtPMR4_F: TGATCGCACAGACTCAAAGG
AtPMR4_R: ATGATGCGAAGTCCTCTGCT
AtPMR5_F: GGCATCATCGTCTTCAGGAT
AtPMR5_R: CTGACGGATGACCGTCTTTT
AtPMR6_F: ACAAGGGCTTGTTCAGAGGA
AtPMR6_R: ATTCACCATCGTCCTTCGAG
AtMyb46_F: GCGCTTTCTCTCCTCAAGAA
AtMyb46_R: TGGGTGATGAGGATGAGTTG
B. *cinerea* -Actin-F: AAGTGTGATGTTGATGTCC
B. *cinerea* -Actin-R: CTGTTGGAAAGTAGACAAAG
B. *cinerea* -PG1-F: TCTTTTACGCCCACTCCTTG
B. *cinerea* -PG1-R: GATATCGCTGGAGGAACCAA
B. *cinerea* -PG2-F: GTGGGATGGACTTGGCTCTA
B. *cinerea* -PG2-R: ACCAGCATCTCCAGCAGAGT
B. *cinerea* -PME3-F: TATCTCTGCCCACACCACAA
B. *cinerea* -PME3-R: ATGGTGCGGATATCGTTGTT
BdPR1-F: ACGGTACGACTACTGGAGCAA
BdPR1-R: GCCGCATGCCGACGTAGTTA

BdPR5-F: ACGGCGTACTCGAAGCTGTT
BdPR5-R: CTACTGAAACACGGTGCCTC
BdbG2-F: GTTCAACGAGAACCAGAAGC
BdbG2-R: AACAGACGATCGATCGATCG
BdPAD3-F: AAGATATCCGGGCACGACGT
BdPAD3-R: AGTCGAAGCTGCACAGTAGG
BdJR1-F: CGGTCCATCACGTTTGTGAG
BdJR1-R: CAGAAGAGGTACTTGGAGAG
BdWR3-F: CCTTCTTCTTCTTCATCGAG
BdWR3-R: CCGAGTATGCCTTGCAATAG
AtActin2_F: GAAACCCTCGTAGATTGGCA
AtActin2_R: CTCTCCCGCTATGTATGTCGC
BdGAPD-F: GCTGGTGCCGATTATGTCGT
BdGAPD-R: AGTGGTGCAGCTAGCATTTGAGACAAT
BdRETO-F: CTAAGATCTTCGCCTGGCTA
BdRETO-R: GCTGACGTACGGTTCCATGA
BdCYP-F: TTGATGCTGCCGTTCGGGAT
BdCYP-R: TTCTGAAGCACGTCACGCAT
BdWRKY-F: CTAATTAATCCCCATGCTCC
BdWRKY-R: TGATCGTCAATCGACCCACT
BdPGIP-F: TCTGGATGTCCCACAACAAC
BdPGIP-R: TTGTGGCTCACGTCGAGGAA
B. *cinerea* -Actin-F: AAGTGTGATGTTGATGTCC
B. *cinerea* -Actin-R: CTGTTGGAAAGTAGACAAAG
B. *sorokiniana*-GDP-F: ATCGAGCCCCACTACGCTGTA
B. *sorokiniana* -GDP-R: TGACGGTCAGGTTGTTGCCGT
